# Optimization of Enzyme-Assisted Ultrasonic Extraction of a Quercetin-Containing Crude Extract from *Sophora japonica* Flower Buds and Evaluation of Its In Vitro Antioxidant and Antibacterial Activities

**DOI:** 10.3390/foods15162781

**Published:** 2026-08-07

**Authors:** Yizhuo Li, Linsen Shao, Xuancheng Guan, Da Qiao, Liyuan Xie, Jing Chen, Xianjun Liu

**Affiliations:** College of Animal Science and Veterinary Medicine, Shenyang Agricultural University, No. 120 Dongling Road, Shenyang 110866, China; 2025220620@stu.syau.edu.cn (Y.L.); 13667383305@163.com (L.S.); 15764380107@163.com (X.G.); 2025240808@stu.syau.edu.cn (D.Q.); 2025220609@stu.syau.edu.cn (L.X.)

**Keywords:** *Sophora japonica*, quercetin, enzyme-assisted extraction, ultrasonic extraction, response surface methodology, HPLC, antioxidant activity, antibacterial activity

## Abstract

Flower buds of *Sophora japonica* are flavonoid-rich edible botanical materials. This study developed an enzyme-assisted ultrasonic protocol for producing a quercetin-containing crude extract. A Box–Behnken design evaluated β-glucosidase concentration, digestion time, and the cellulase-to-pectinase mass ratio. The quadratic model was significant (*p* < 0.0001; *R*^2^ = 0.9848; adjusted *R*^2^ = 0.9652). At 14 U/mL β-glucosidase, 72 min of digestion, and a mass ratio of 1.25:1 (*w*/*w*), the UV-estimated quercetin-equivalent yield reached 7.34%, compared with 4.17% for ultrasound alone (*p* < 0.001). HPLC based on retention-time agreement and external calibration estimated quercetin at 71.93 ± 1.35 mg/g initial dry plant material. The dried extract contained 82.93 ± 3.75 mg gallic acid equivalents/g and 154.68 ± 5.56 mg quercetin equivalents/g. It showed concentration-dependent DPPH^•^, ^•^OH, and ABTS^•+^ scavenging, with IC_50_ values of 1.339 ± 0.061, 3.931 ± 0.108, and 1.004 ± 0.032 mg/mL, respectively, and preliminary antibacterial activity against *Salmonella enterica*, *Escherichia coli*, and *Staphylococcus aureus*. These findings establish a defined extraction route while distinguishing the UV optimization endpoint from HPLC-based quercetin estimation.

## 1. Introduction

Edible botanical materials are widely investigated as sources of flavonoids and related phytochemicals. Quercetin, one of the best-studied flavonols, has been associated with redox-related and antimicrobial effects in experimental systems [1,2].

Dried flower buds of *Sophora japonica* are used as an edible and medicinal botanical resource [3] and are particularly rich in flavonols. According to the United States Pharmacopeia–National Formulary (USP–NF) monograph, they contain 14–27% total flavonol glycosides on a dry-weight basis, consisting mainly of rutin (13–25%), together with kaempferol-3-*O*-rutinoside and isorhamnetin-3-*O*-rutinoside; free quercetin is specified at 0.3–1.0% [4]. These ranges provide a quantitative compositional benchmark rather than a batch-specific characterization of the material used in the present study, because the flavonoid profile of *S. japonica* may vary with flower maturity [5]. Although rutin is the predominant native flavonoid, quercetin was selected as the analytical target because it is the aglycone of rutin and has been extensively investigated for its antioxidant and antibacterial properties [1,2].

In dried plant material, solvent access to these components can be constrained by the tissue matrix, making conventional extraction comparatively slow. Conventional solvent extraction typically depends on prolonged solvent–solid contact and may require heating and relatively large solvent volumes [6]. Ultrasound is commonly applied to plant materials because cavitation can improve solvent contact with the tissue matrix. For *S. japonica* flower buds, ultrasound-assisted methanolic extraction has been reported to shorten extraction time and increase the maximum rutin yield compared with conventional methanolic extraction [7]. Enzymes directed against structural polysaccharides may further improve access to intracellular components. Combining the two steps has therefore been explored for recovering phytochemicals from botanical materials [6,8]. Through the complementary effects of cell-wall degradation and cavitation-enhanced mass transfer, enzyme-assisted ultrasonication may further improve extraction efficiency. However, enzymatic pretreatment requires enzymes, buffer, and additional digestion time. Its overall sustainability should therefore be evaluated in terms of extraction yield, solvent and water consumption, energy demand, processing time, and downstream handling rather than inferred from extraction efficiency alone.

The influence of extraction factors is rarely independent. Response surface methodology (RSM) provides a unified experimental framework in which linear effects, pairwise interactions, and response curvature can be evaluated simultaneously, rather than by a sequence of separate one-factor experiments [9]. Previous studies on *S. japonica* flower buds have compared conventional and ultrasound-assisted extraction of rutin [7], optimized ultrasound-assisted extraction of six individual flavonoids and total flavonoids while evaluating antioxidant activity [10], and investigated subcritical-water extraction of rutin and quercetin [11]. Unlike these earlier studies, the present work first established an optimized ultrasound-only procedure as a nonenzymatic reference and then optimized an enzyme-assisted procedure by varying β-glucosidase concentration, digestion time, and the cellulase-to-pectinase mass ratio. It also explicitly distinguished the UV-estimated quercetin-equivalent yield used for process optimization from the HPLC-based estimation of quercetin and further characterized the dried crude extract in terms of total phenolic and flavonoid contents and in vitro antioxidant and antibacterial activities.

Accordingly, this work optimized enzyme-assisted ultrasonic extraction of *Sophora japonica* flower buds and characterized the resulting crude extract. The primary objective was to determine whether enzymatic pretreatment followed by ultrasonication could increase the UV-estimated quercetin-equivalent yield relative to the optimized ultrasound-only procedure. Quercetin was estimated by HPLC, while total phenolic and flavonoid contents were determined by colorimetric assays. Three radical-scavenging assays and the ferric reducing antioxidant power (FRAP) assay were used to assess antioxidant responses, and antibacterial testing was conducted against *Salmonella enterica*, *Escherichia coli*, and *Staphylococcus aureus*.

## 2. Materials and Methods

### 2.1. Plant Materials, Reagents, and Microbial Strains

Dried *S. japonica* flower buds were purchased from Guangdong Fudonghai Pharmaceutical Co., Ltd. (Zhanjiang, China), milled, and stored under dry conditions. Quercetin reference material was obtained from Solarbio Science & Technology Co., Ltd. (Beijing, China; batch no. SQ8030). β-Glucosidase (14 U/mg; batch no. G824159), cellulase (10,000 U/g; batch no. C805042), and pectinase (10,000 U/g; batch no. P816736) were from Macklin Biochemical Co., Ltd. (Shanghai, China). HPLC-grade methanol (catalog no. 10014118), analytical-grade phosphoric acid (catalog no. 10015418), absolute ethanol (catalog no. 10009218), aluminum chloride, sodium nitrite, and sodium hydroxide were obtained from Sinopharm Chemical Reagent Co., Ltd (Shanghai, China).

Folin–Ciocalteu reagent (F8060), gallic acid (SG8040; ≥98%), 2,2-diphenyl-1-picrylhydrazyl (DPPH; ID1240; ≥97%), a 2,2′-azino-bis(3-ethylbenzothiazoline-6-sulfonic acid) (ABTS) assay kit (BC4245), a ferric reducing antioxidant power (FRAP) assay kit (BC1310), vitamin C (A8100; ≥99%), enrofloxacin (E8761; ≥98%), nutrient broth (N8030), nutrient agar (A8190), Mueller–Hinton agar (M8480), and Mueller–Hinton broth (MHB; M8556) were purchased from Solarbio (Beijing, China). The hydroxyl radical (^•^OH) assay kit (JL-T0042) was obtained from Jianglai Biotech (Shanghai, China). *E. coli* BNCC133264, *S. enterica* BNCC108207, and *S. aureus* BNCC186335 were supplied by the BeNa Culture Collection (Beijing, China).

The principal equipment included an Agilent 1260 Infinity HPLC instrument with diode-array detection (Agilent Technologies, Santa Clara, CA, USA), UV-5100 UV–visible spectrophotometer (METASH, Shanghai, China), SB-5200DT ultrasonic cleaner (Scientz, Ningbo, China; 360 W, 40 kHz), HHS-11-4 water bath (Boxun, Shanghai, China), TDZ4-WS centrifuge (Xiangyi, Changsha, China), Synergy HT plate reader (BioTek Instruments, Inc., Winooski, VT, USA), SPX-250B-Z incubator (Boxun, Shanghai, China), BS210S analytical balance (Sartorius, Göttingen, Germany), RHPH-500A grinder (Rongsheng, Hangzhou, China), DHG-9030A drying oven (Yiheng, Shanghai, China), and HZQOLB shaking incubator (Huahong, Wuhan, China).

### 2.2. Determination of UV-Estimated Quercetin-Equivalent Yield

The RSM response was expressed as a UV-estimated quercetin-equivalent yield. Absorbance at 374 nm was converted using the quercetin calibration equation *A* = 0.0589*C* + 0.0599 (*R*^2^ = 0.9998), where *A* is absorbance and *C* is the quercetin standard concentration (μg/mL). The sample quercetin-equivalent concentration, *C_s_*, was obtained from the same calibration equation. The UV-estimated quercetin-equivalent yield was calculated using Equation (1):

(1) $$Y_{UV}\left(\%\right)\;=\frac{C_{S}VD}{10^{4}m}$$

where *C_s_* is the quercetin-equivalent concentration of the measured sample solution (μg/mL), *V* is the total volume of the original extract before dilution (mL), *D* is the total dilution factor applied before absorbance measurement, and *m* is the initial dry mass of plant material (g). The factor 10^4^ converts μg/g to percent because 1% (*w*/*w*) is equivalent to 10,000 μg/g. Thus, the UV-derived concentration was not reported directly in mg/mL but was converted to a quercetin-equivalent mass fraction expressed as % (*w*/*w*) of initial dry plant material. Because the samples were crude extracts, absorbance at 374 nm could include contributions from quercetin and other co-extracted UV-absorbing constituents, including flavonoids and other phenolic compounds. Accordingly, the UV-derived result was expressed as a quercetin-equivalent yield and used to compare extraction conditions rather than as compound-specific quantification of quercetin. The recovered supernatants obtained under the optimized extraction condition were subsequently assessed by targeted HPLC.

### 2.3. Ultrasound-Only Extraction and Comparative Optimization

Preliminary one-factor experiments were used only to select factor ranges for the ultrasound-only extraction RSM. The three numerical factors were ethanol concentration, ultrasonication time, and liquid-to-material ratio. The Box–Behnken matrix, model ANOVA, and response surfaces are provided in Tables S1 and S2 and Figure S1.

For ultrasound alone, the validated operating point was 68% ethanol, 14 min of ultrasonication, and 19 mL/g liquid-to-material ratio. The corresponding UV-estimated quercetin-equivalent yield was 4.17%; this condition served as the nonenzymatic comparator.

### 2.4. Enzyme-Assisted Ultrasonic Extraction and Box–Behnken Design

The enzyme-assisted design varied β-glucosidase concentration, digestion time, and the cellulase-to-pectinase mass ratio. The factor ranges were selected primarily on practical grounds to span low, intermediate, and high β-glucosidase inputs and pretreatment durations that were feasible within the laboratory workflow. Specifically, β-glucosidase concentration and digestion time were varied from 6 to 14 U/mL and from 50 to 90 min, respectively, with center points of 10 U/mL and 70 min. The cellulase-to-pectinase mass ratio was varied from 0.5 to 2.0, with a center point of 1.25, to cover pectinase-rich, intermediate, and cellulase-rich formulations while keeping the combined mass of the two commercial enzyme preparations constant, thereby avoiding confounding between enzyme proportion and total enzyme input.

Each run started with 10.0 g of *S. japonica* flower-bud powder. A 50 mL cellulase–pectinase mixture and 100 mL β-glucosidase solution were added. The cellulase-to-pectinase ratio was calculated from the masses of the commercial enzyme preparations, with cellulase as the numerator and pectinase as the denominator. The combined mass of the cellulase and pectinase preparations was fixed at 50.0 mg in 50 mL of buffer. Both commercial preparations had nominal activities of 10,000 U/g; however, the experimental ratio was defined strictly on a mass basis. Accordingly, a ratio of 1.25 represented a cellulase-to-pectinase mass ratio of 1.25:1 (*w*/*w*); under this condition, 27.8 mg of the cellulase preparation and 22.2 mg of the pectinase preparation were used per 10.0 g of plant material. β-Glucosidase had a nominal activity of 14 U/mg; therefore, preparation of 100 mL solutions at 6, 10, or 14 U/mL required 42.9, 71.4, or 100.0 mg of the enzyme, respectively. Under the validated conditions, the total enzyme mass was 150.0 mg in 150 mL of 0.1 M acetate buffer prepared from acetic acid and sodium acetate at pH 5.0.

Digestion was performed at 37 °C for the assigned duration. The mixture was then centrifuged at 4000 rpm for 10 min, and the liquid phase was discarded. The retained solids were ultrasonicated with 190 mL of 68% (*v*/*v*) ethanol, equivalent to 19 mL/g initial dry plant material, at 360 W and 40 kHz for 14 min at approximately 25 °C. After a second centrifugation at 4000 rpm for 10 min, all recovered supernatant was quantitatively collected. Because the recovered volume was below 150 mL for each batch, it was adjusted to a final volume of 150 mL with 68% (*v*/*v*) ethanol. This standardized volume represented the total recovered supernatant for each extraction batch and was used for the subsequent HPLC-based content calculation. An aliquot of the volume-adjusted recovered supernatant was reserved for HPLC analysis, and the remaining supernatant from each batch was concentrated by rotary evaporation at 40 °C, dried to constant mass, and weighed. Portions of each batch were then accurately weighed to prepare the TPC, TFC, antioxidant, and antibacterial assay solutions; the concentrations reported for these assays therefore refer to dried crude extract mass.

The Box–Behnken design contained seventeen runs, five of which were center-point replicates. The following second-order model was fitted to the response:

(2) $$Y\;=\;\beta_{0}\;+\;\beta_{1}A\;+\;\beta_{2}B\;+\;\beta_{3}C\;+\;\beta_{12}AB\;+\;\beta_{13}AC\;+\;\beta_{23}BC\;+\;\beta_{11}A^{2}\;+\;\beta_{22}B^{2}\;+\;\beta_{33}C^{2}$$

Here, *Y* denotes the predicted UV-estimated quercetin-equivalent yield. *β*_0_ is the intercept; *β*_1_–*β*_3_ are linear terms; *β*_12_–*β*_23_ are two-factor interactions; and *β*_11_–*β*_33_ are quadratic terms. For *A*, *B*, and *C*, coded levels of −1, 0, and +1 represented the low, center, and high settings. The coded equation obtained was:

(3) $$\begin{array}{c} Y\;=\;7.2920\;+\;0.1988A\;+\;0.0688B\;+\;0.0050C\;-\;0.0025AB\;+\;0.0250AC\\ -\;0.0550BC\;-\;0.1323A^{2}\;-\;0.3673B^{2}\;-\;0.2998C^{2} \end{array}$$

Model performance was judged from ANOVA, *R*^2^, adjusted *R*^2^, predicted *R*^2^, coefficient of variation (C.V.), and the lack-of-fit test.

### 2.5. HPLC Quantification of Quercetin

Quercetin in the recovered supernatants was assessed by targeted HPLC. External standards between 10 and 100 μg/mL were used to construct the peak-area calibration: *y* = 42,495.1*x* + 9012.9 (*R*^2^ = 0.9999997), where *y* is peak area and *x* is the quercetin concentration in the injected solution (μg/mL).

For HPLC quantification, three independent extraction batches were prepared under the validated enzyme-assisted ultrasonic extraction protocol, with each batch starting from 10.0 g of flower-bud powder. For each batch, all supernatant recovered after the second centrifugation was quantitatively collected. Because the recovered volume was below 150 mL, it was adjusted to a final volume of 150 mL with 68% (*v*/*v*) ethanol. A 1.5 mL aliquot of the volume-adjusted recovered supernatant was diluted to a final volume of 100 mL, corresponding to a dilution factor of 66.67, and filtered through a 0.22 μm membrane. A 10 μL portion of each filtrate was injected once. The three samples were analyzed in the same analytical sequence as the calibration standards under the chromatographic conditions described below, and identical integration parameters were applied to all chromatograms. Each independently prepared extraction batch constituted one experimental unit.

The quercetin concentration in each injected solution was calculated from the calibration equation and expressed in μg/mL. The concentration in the corresponding recovered supernatant was calculated by multiplying the injected-solution concentration by the 66.67-fold dilution factor and dividing by 1000 to convert μg/mL to mg/mL. Quercetin content relative to the initial dry plant material was then calculated using the recovered-supernatant concentration, the standardized total recovered-supernatant volume of 150 mL, and the initial dry plant mass of 10.0 g, and was reported as mg/g initial dry plant material. Batch-specific values were calculated separately and subsequently summarized as mean ± SD for the three independent extraction batches (*n* = 3).

Separation involved an Agilent 1260 Infinity HPLC system with diode-array detection and a Diamonsil C18 column (4.6 mm × 250 mm, 5 μm; Dikma Technologies, Beijing, China; catalog no. 99903). The mobile phase was methanol/0.4% phosphoric acid (60:40, *v*/*v*) under isocratic conditions at 0.8 mL/min. The column was kept at 25 °C; injection volume was 10 μL; detection was at 370 nm.

### 2.6. Determination of Total Phenolic Content and Total Flavonoid Content

TPC was determined separately for each of the three independently prepared dried optimized crude extract batches using the Folin–Ciocalteu assay [12,13], with gallic acid as the reference standard (Solarbio, Beijing, China; SG8040). For each batch, 100 mg of dried crude extract was dissolved in distilled water and brought to a final volume of 10 mL. Before color development, the prepared sample solution was diluted 50-fold. A 100 μL aliquot of the diluted sample solution was mixed with 1.0 mL of Folin–Ciocalteu reagent previously diluted 10-fold with distilled water and 3.0 mL of 7.5% (*w*/*v*) Na_2_CO_3_ solution. The reaction mixture was incubated in the dark at room temperature for 30 min, and absorbance was measured at 760 nm using a UV–visible spectrophotometer. Gallic acid standards and sample solutions were subjected to the same color-development procedure, and distilled water was used in place of the standard or sample solution for the reagent blank. The calibration range was 0–18 μg/mL, with the equation *A* = 0.032262*C*_GA_ − 0.0012 (*R*^2^ = 0.9999), where *A* is absorbance and *C*_GA_ is the calibration-derived gallic acid-equivalent concentration of the 50-fold-diluted sample solution (μg/mL). TPC was calculated using Equation (4):

(4) $$TPC\left(mg\;GAE/g\right)\;=\frac{C_{GA}VD}{1000m}$$

where *V* is the initial volume of the prepared crude extract solution (10 mL), *D* is the total pre-assay dilution factor (50), and *m* is the mass of dried crude extract used to prepare the solution (0.100 g). Results were expressed as mg gallic acid equivalents per g of dried crude extract.

TFC was determined separately for each of the three independently prepared dried optimized crude extract batches using the aluminum chloride colorimetric method [13], with quercetin as the reference standard. For each batch, 50 mg of dried crude extract was dissolved in 70% (*v*/*v*) ethanol and brought to a final volume of 10 mL. Before color development, the prepared sample solution was diluted 200-fold. A 1.0 mL aliquot of the diluted sample solution was mixed with 0.3 mL of 5% (*w*/*v*) NaNO_2_ solution and allowed to react for 6 min. Subsequently, 0.3 mL of 10% (*w*/*v*) AlCl_3_ solution was added, followed by a further 6 min reaction. After the addition of 2.0 mL of 1 M NaOH, the reaction mixture was brought to a final volume of 10 mL with distilled water, incubated at room temperature for 15 min, and measured at 510 nm using a UV–visible spectrophotometer. Quercetin standards and sample solutions were subjected to the same color-development procedure, and 70% (*v*/*v*) ethanol was used in place of the standard or sample solution for the reagent blank. The calibration range was 0–7 μg/mL, with the equation *A* = 0.0699*C*_QE_ − 0.0243 (*R*^2^ = 0.9981), where *A* is absorbance and *C*_QE_ is the calibration-derived quercetin-equivalent concentration of the 200-fold-diluted sample solution (μg/mL). TFC was calculated using Equation (5):

(5) $$TFC\left(mg\;QE/g\right)\;=\frac{C_{QE}VD}{1000m}$$

where *V* is the initial volume of the prepared crude extract solution (10 mL), *D* is the total pre-assay dilution factor (200), and *m* is the mass of dried crude extract used to prepare the solution (0.050 g). Results were expressed as mg quercetin equivalents per g of dried crude extract.

### 2.7. Antioxidant Activity Assays

Each of the three independently prepared dried optimized crude extract batches was evaluated alongside vitamin C in DPPH^•^, ^•^OH, ABTS^•+^, and FRAP assays [14,15,16]. For each batch, the dried crude extract and vitamin C were tested at 0.25, 0.5, 0.75, 1, 1.5, 2, 3, 4, 5, 6, 8, and 10 mg/mL; extract concentrations refer to the mass of dried crude extract in solution. In the DPPH^•^ assay, 100 μL sample or vitamin C solution in absolute ethanol was combined with 100 μL 0.1 mM DPPH^•^ in absolute ethanol in a 96-well plate. After 30 min at room temperature in the dark, absorbance was read at 517 nm. The DPPH^•^ control contained ethanol plus DPPH^•^; sample blanks used ethanol in place of DPPH^•^. ^•^OH scavenging activity was measured at 510 nm using the Jianglai kit (JL-T0042; Jianglai Biotech, Shanghai, China). ABTS^•+^ scavenging activity and FRAP were measured at 734 and 593 nm, respectively, using Solarbio kits BC4245 and BC1310 (Solarbio, Beijing, China), as directed by the manufacturer. FRAP results are reported as μmol FeSO_4_ equivalents/mL.

For DPPH^•^, ^•^OH, and ABTS^•+^ scavenging assays, scavenging activity was calculated as follows:

(6) $$Scavenging\;activity\left(\%\right)=\left[1\;-\;\frac{A_{sample}\;-\;A_{blank}}{A_{control}}\right]\times 100$$

For each radical-scavenging assay, *A*_sample_ was the absorbance of the sample reaction, *A*_blank_ was the corresponding color blank, and *A*_control_ was the reaction without sample. The radical reagent was replaced with the relevant solvent in blank wells to correct for the inherent color of the extract. For each of the DPPH^•^, ^•^OH, and ABTS^•+^ assays, the concentration-response dataset from each independent dried crude extract batch was fitted separately using a two-parameter Hill model, with the lower and upper asymptotes constrained to 0% and 100%, respectively:

(7) $$Y\;=\frac{100C^{h}}{IC_{50}^{h}+C^{h}}$$

where *Y* is the radical-scavenging activity (%), *C* is the tested concentration (mg/mL), IC_50_ is the concentration producing 50% scavenging, and *h* is the Hill slope. The three corresponding vitamin C datasets were fitted separately using the same model. Batch- or run-specific IC_50_ values were subsequently summarized as mean ± SD (*n* = 3).

### 2.8. Antibacterial Activity Assays

Antibacterial testing used *S. enterica* BNCC108207, *E. coli* BNCC133264, and *S. aureus* BNCC186335. Fresh cultures were prepared on agar slants at 37 °C for 18–24 h. Nutrient agar and nutrient broth were used for routine culture, while Mueller–Hinton agar (MHA) was used for disk diffusion. Bacterial suspensions were adjusted to 0.5 McFarland, approximately 1 × 10^8^ CFU/mL, and 100 μL was spread over each MHA plate. For each independently prepared dried crude extract batch, 3.0, 6.0, and 10.0 mg of the dried optimized crude extract were separately weighed, dissolved in a small volume of absolute ethanol with vortex mixing and sonication to facilitate dissolution, quantitatively transferred to separate 100 mL volumetric flasks, and diluted to volume with absolute ethanol. The resulting loading solutions contained 30, 60, and 100 μg/mL dried crude extract, respectively. Sterile 6 mm disks were loaded with 10 μL of each solution, corresponding to 0.3, 0.6, and 1.0 μg of dried crude extract per disk, respectively. Enrofloxacin solution (10 μg/mL; 10 μL/disk, corresponding to 0.1 μg/disk) served as the positive control, whereas absolute ethanol (10 μL/disk) served as the vehicle control. Loaded disks were dried at 37 °C for 24–36 h, placed onto inoculated MHA, and incubated for 24 h at 37 °C. Inhibition-zone diameters were measured, including the 6 mm disk diameter. Disk-diffusion assays were independently performed using each of the three dried crude extract batches, and each independently prepared batch was treated as one experimental unit.

MIC and MBC were measured by broth microdilution in 96-well plates and are reported as μg/mL dried crude extract. For each independently prepared dried crude extract batch, a 100 mg/mL stock solution was prepared by dissolving 100 mg of the corresponding dried crude extract in 1 mL of absolute ethanol. Two-fold dilutions were prepared in MHB at twice the intended final concentration. Each well received 100 μL diluted extract and 100 μL bacterial suspension at approximately 1 × 10^6^ CFU/mL. The final volume was 200 μL, with 0.5–512 μg/mL crude extract and approximately 5 × 10^5^ CFU/mL bacteria. At 512 μg/mL, final ethanol was 0.512% (*v*/*v*) and declined two-fold across subsequent concentrations. For every organism, a matched 0.512% ethanol vehicle control, a growth control, and a sterility control were included. After 24 h at 37 °C, the MIC was the lowest concentration without visible growth. OD_600_ was measured for controls. A 10 μL aliquot from each clear well was transferred onto nutrient agar and incubated at 37 °C for 24 h; the lowest concentration yielding no colonies was recorded as the MBC. The MBC/MIC ratio was used only as a preliminary description of antibacterial behavior. Broth-microdilution assays were independently performed using each of the three dried crude extract batches, and each independently prepared batch was treated as one experimental unit.

### 2.9. Statistical Analysis

Design-Expert 13 software (Stat-Ease, Inc., Minneapolis, MN, USA) was used to fit the RSM models. ANOVA was used to evaluate overall model significance, individual model terms, and lack of fit. Where group comparisons were applicable, non-RSM continuous data were analyzed using IBM SPSS Statistics 20.0 (IBM Corp., Armonk, NY, USA) with one-way ANOVA followed by Duncan’s multiple-range test. For HPLC quantification, TPC, TFC, antioxidant assays, disk-diffusion assays, and broth-microdilution assays, three independent extraction batches were prepared under the validated enzyme-assisted ultrasonic extraction protocol. Each independently prepared batch constituted one experimental unit. HPLC analysis was performed with one injection per batch. For measurements of the dried crude extract, continuous data are presented as mean ± SD for three independent extraction batches (*n* = 3). Vitamin C and enrofloxacin were assayed in parallel in the corresponding three analytical runs, and their data are also presented as mean ± SD (*n* = 3). For each radical-scavenging assay, concentration-response data from each independent dried crude extract batch and each corresponding vitamin C analytical run were fitted separately using the constrained two-parameter Hill model described in Section 2.7. The resulting IC_50_ values are presented as mean ± SD (*n* = 3). Nonlinear regression analyses for IC_50_ estimation were performed using OriginPro 2026 (version 10.3; OriginLab Corporation, Northampton, MA, USA). MIC and MBC were determined separately for each independent dried crude extract batch and are reported as the common endpoint when identical across all three batches. A value of *p* < 0.05 was considered statistically significant.

## 3. Results and Discussion

### 3.1. Optimization of Enzyme-Assisted Ultrasonic Extraction

The 17 combinations and their UV-estimated quercetin-equivalent yields are listed in Table 1. Values ranged from 6.45% to 7.33%. The higher values occurred close to the center of the factor space, whereas moving away from those conditions reduced the measured yield.

The quadratic model was significant (*F* = 50.30, *p* < 0.0001; Table 2). Lack of fit was not significant (*p* = 0.0781), and the model explained most of the observed variation (*R*^2^ = 0.9848; adjusted *R*^2^ = 0.9652). The predicted *R*^2^ (0.8030) was lower than the adjusted *R*^2^, indicating that predictive performance was weaker than the within-design fit. However, the difference between the adjusted and predicted *R*^2^ values was 0.1622, indicating reasonable agreement between model fit and prediction. Together with the low C.V. (0.83%), these results support the use of the model for interpolation and optimization within the tested factor space, whereas extrapolation beyond the studied ranges should be avoided.

**Figure 1:**
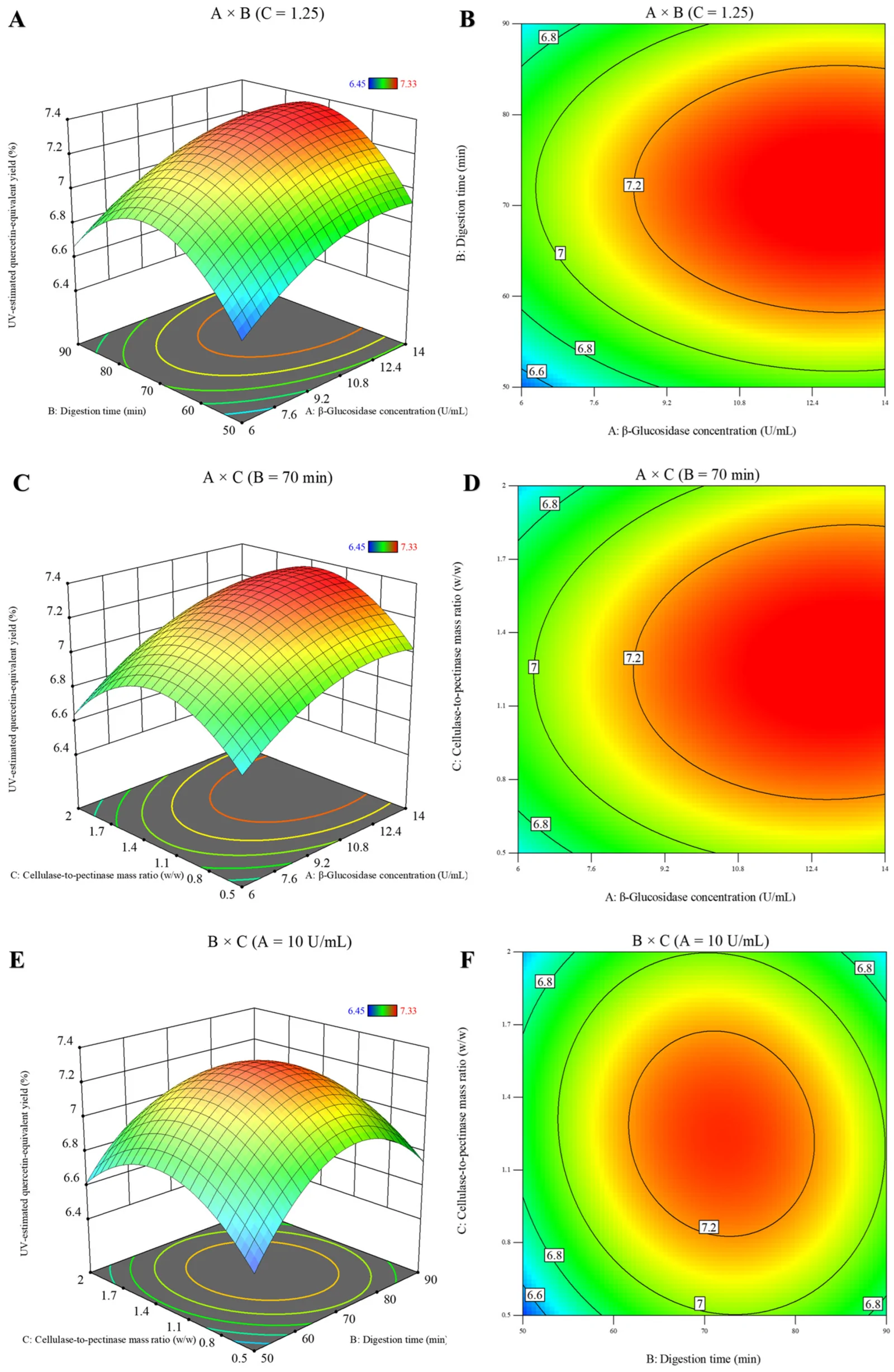
Model-based response surfaces (**A**,**C**,**E**) and contour plots (**B**,**D**,**F**) for enzyme-assisted ultrasonic extraction. Panels (**A**,**B**) show β-glucosidase concentration and digestion time when *C* = 1.25, corresponding to a cellulase-to-pectinase mass ratio of 1.25:1 (*w*/*w*). Panels (**C**,**D**) show β-glucosidase concentration and the cellulase-to-pectinase mass ratio at 70 min. Panels (**E**,**F**) show digestion time and the cellulase-to-pectinase mass ratio at 10 U/mL β-glucosidase. The response plotted is UV-estimated quercetin-equivalent yield (%).

Among the linear terms, β-glucosidase concentration (A) and digestion time (B) contributed significantly, whereas the linear term for the cellulase-to-pectinase mass ratio (C) did not. Significant A^2^, B^2^, and C^2^ terms indicate curvature in the response surface. The AB, AC, and BC interactions were not significant; Figure 1 is therefore interpreted primarily as a depiction of the fitted response region rather than as evidence for strong pairwise interactions.

Within the tested factor space, the model predicted the maximum response at 14 U/mL β-glucosidase, 71.75 min of digestion, and a cellulase-to-pectinase mass ratio of 1.28:1 (*w*/*w*), with an estimated yield of 7.36%. For validation, the selected settings were 14 U/mL β-glucosidase, 72 min of digestion, and a cellulase-to-pectinase mass ratio of 1.25:1 (*w*/*w*). The measured yield was 7.34%, corresponding to a relative error of 0.30% from the predicted value (Table 3). This close agreement supports the adequacy of the model at the selected validation point.

Relative to the validated ultrasound-only process, the enzyme-assisted procedure increased the UV-estimated quercetin-equivalent yield from 4.17 ± 0.22% to 7.34 ± 0.45%, corresponding to a relative increase of approximately 76.0% (*p* < 0.001; Table 3). Published studies provide useful context for this improvement, but direct quantitative comparison is limited by differences in botanical matrices, target analytes, extraction procedures, analytical methods, and reporting units.

For *S. japonica* flower buds, Liao et al. reported a rutin yield of 153.93 ± 9.41 mg/g using Soxhlet extraction and 182.25 ± 13.38 mg/g using their optimized ultrasound-assisted procedure, corresponding to an increase of approximately 18.4% under their experimental conditions [17]. In other flavonoid-rich botanical materials, Ghasemzadeh et al. reported an optimized quercetin yield of 0.394 mg/g on a dry-weight basis from *Murraya koenigii* leaves [18], whereas El Maaiden et al. reported a maximum isoquercetin recovery of 1033.96 ± 3.28 μg/g from *Ephedra alata* [19]. These studies used chromatographic, analyte-specific endpoints, whereas the present study used a UV-estimated quercetin-equivalent yield expressed as a percentage of initial dry plant material. Accordingly, these values should not be directly ranked against the present UV-estimated quercetin-equivalent yield; rather, they illustrate the strong dependence of reported extraction performance on botanical matrix, target flavonoid, extraction conditions, and analytical basis.

The higher UV-estimated quercetin-equivalent yield observed in the present study may reflect both enhanced matrix accessibility and enzyme-mediated changes. Cellulase and pectinase may hydrolyze structural cell-wall polysaccharides and disrupt the retained solid matrix, thereby improving solvent access during subsequent ethanolic ultrasonication [20]. β-Glucosidase may also act on susceptible flavonoid β-glucosides and alter the profile of flavonoid compounds contributing to the UV-derived signal [21]. Because the relative contributions of these mechanisms were not resolved analytically, the observed increase should not be attributed solely to improved matrix accessibility.

### 3.2. HPLC-Based Assessment and Phytochemical Characterization

**Figure 2:**
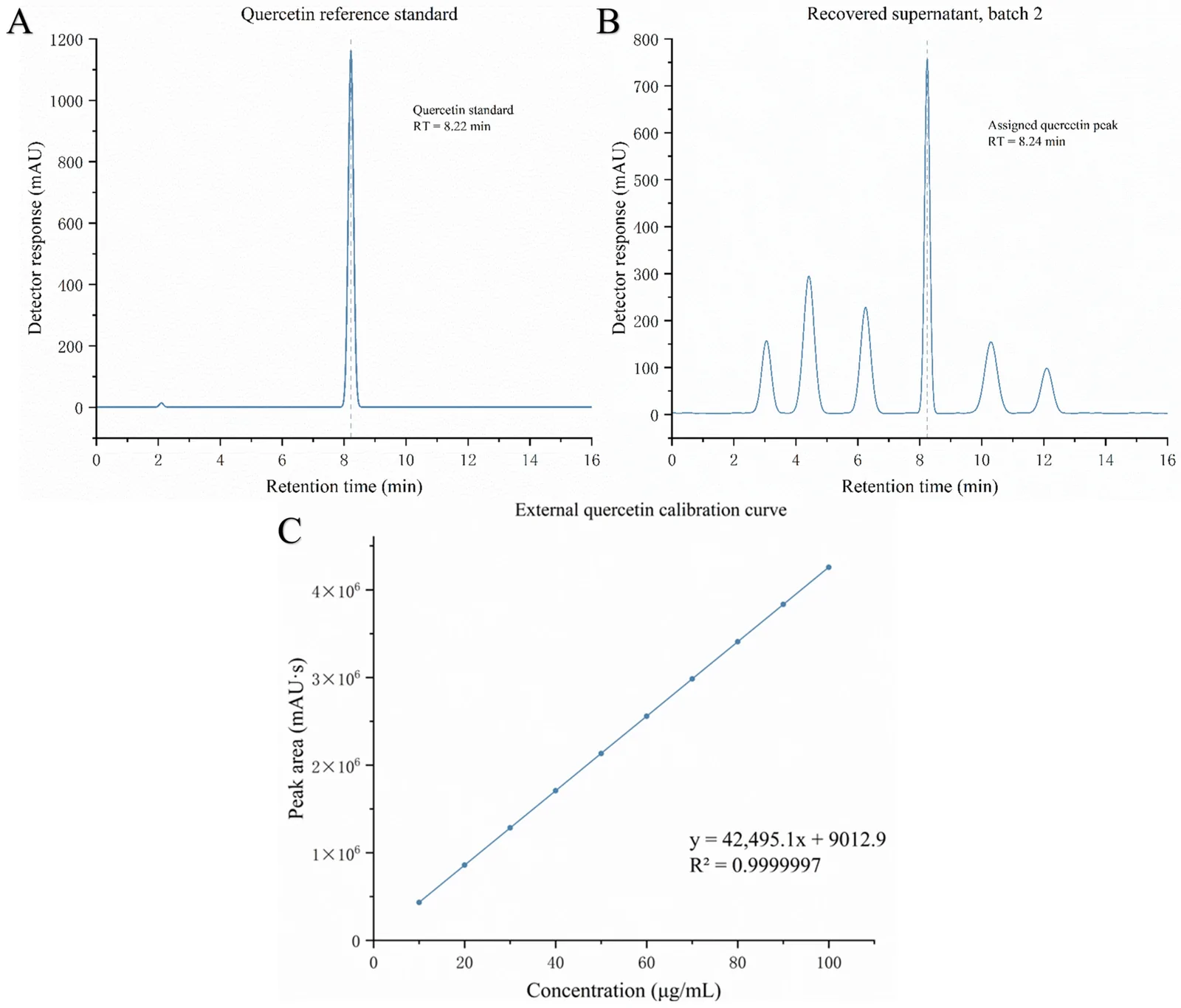
HPLC-based assessment of quercetin in recovered supernatants obtained under the optimized extraction condition. (**A**) Chromatogram of the quercetin reference standard. (**B**) Representative chromatogram of the recovered supernatant from extraction batch 2, one of three independently prepared batches obtained under the optimized conditions. The vertical dashed lines in (**A**,**B**) indicate the retention times of the quercetin reference standard (8.22 min) and the assigned quercetin peak in extraction batch 2 (8.24 min), respectively. (**C**) External peak-area calibration curve for quercetin over the range of 10–100 μg/mL. The assigned peak in the representative sample from extraction batch 2 was attributed to quercetin based on retention-time agreement with the reference standard (8.24 min versus 8.22 min), whereas quantification was performed using the external calibration curve shown in (**C**). Across the three independent extraction batches, the retention times of the assigned peaks ranged from 8.23 to 8.24 min, and quantitative HPLC results are reported as mean ± SD (*n* = 3). Full chromatograms for all three independent extraction batches and an overlay of the assigned quercetin peak region are provided in Figure S2.

Targeted HPLC was used to estimate quercetin in the recovered supernatants from the three independently prepared optimized extraction batches. The quercetin standard eluted at 8.22 min, whereas the representative peak from extraction batch 2 eluted at 8.24 min (Figure 2A,B). The external peak-area calibration was linear over 10–100 μg/mL (*y* = 42,495.1*x* + 9012.9; *R*^2^ = 0.9999997; Figure 2C).

Assigned peaks in extraction batches 1–3 eluted at 8.23–8.24 min and closely agreed with the quercetin reference standard at 8.22 min (Figure 2 and Figure S2). The overlaid peak regions also showed close chromatographic agreement among batches. The mean peak area was 3,065,654 ± 57,187, corresponding to a quercetin concentration of 71.93 ± 1.35 μg/mL in the injected solutions. After correction for the 66.67-fold dilution, the concentration in the standardized recovered supernatant was 4.795 ± 0.090 mg/mL. Using the standardized total recovered-supernatant volume of 150 mL and the initial dry plant mass of 10.0 g per batch, the HPLC-estimated quercetin content was 71.93 ± 1.35 mg/g initial dry plant material, equivalent to 7.19 ± 0.13% (Table 4). This basis differs from that used for the dried crude extract and should not be interpreted as quercetin per g dry extract. The proximity of the UV and HPLC estimates is useful as a consistency check for the optimized extraction condition, but it does not convert the UV response into chromatographic quantification for each design point.

Rutin is the predominant native flavonol glycoside in *S. japonica* flower buds, whereas quercetin is its aglycone and generally occurs at substantially lower native concentrations [4]. Because glycosylation affects flavonoid polarity and solubility [21], rutin and quercetin may exhibit different recoveries under different processing and extraction conditions. Previous studies have reported both rutin and quercetin in processed or extracted materials from *S. japonica* [11,22,23]. In the present workflow, the HPLC-estimated quercetin may reflect improved release of pre-existing quercetin, differential partitioning during enzymatic pretreatment and subsequent ethanolic extraction, possible deglycosylation of susceptible flavonoid glycosides, or a combination of these effects. However, rutin is quercetin-3-*O*-rutinoside, and its complete conversion to quercetin would require cleavage of both the rhamnosyl and glucosyl linkages; the inclusion of β-glucosidase in the protocol alone does not demonstrate that this conversion occurred [21]. Because rutin, intermediate quercetin glycosides, and quercetin were not quantified before and after enzymatic pretreatment or separately in the discarded pretreatment liquid and the retained solid fraction, the present data cannot distinguish enhanced extractability from enzyme-mediated conversion. Accordingly, the HPLC result should be interpreted as an estimate of quercetin in the recovered supernatant rather than as direct evidence of rutin-to-quercetin conversion.

TPC and TFC of the dried optimized crude extract were 82.93 ± 3.75 mg GAE/g and 154.68 ± 5.56 mg QE/g, respectively (Table 4). These colorimetric indices are normalized to dried crude extract mass, whereas the HPLC value is normalized to initial dry plant material. They are therefore complementary descriptions of the product, not directly interchangeable numerical measures.

### 3.3. Antioxidant Activity of the Dried Optimized Crude Extract

**Figure 3:**
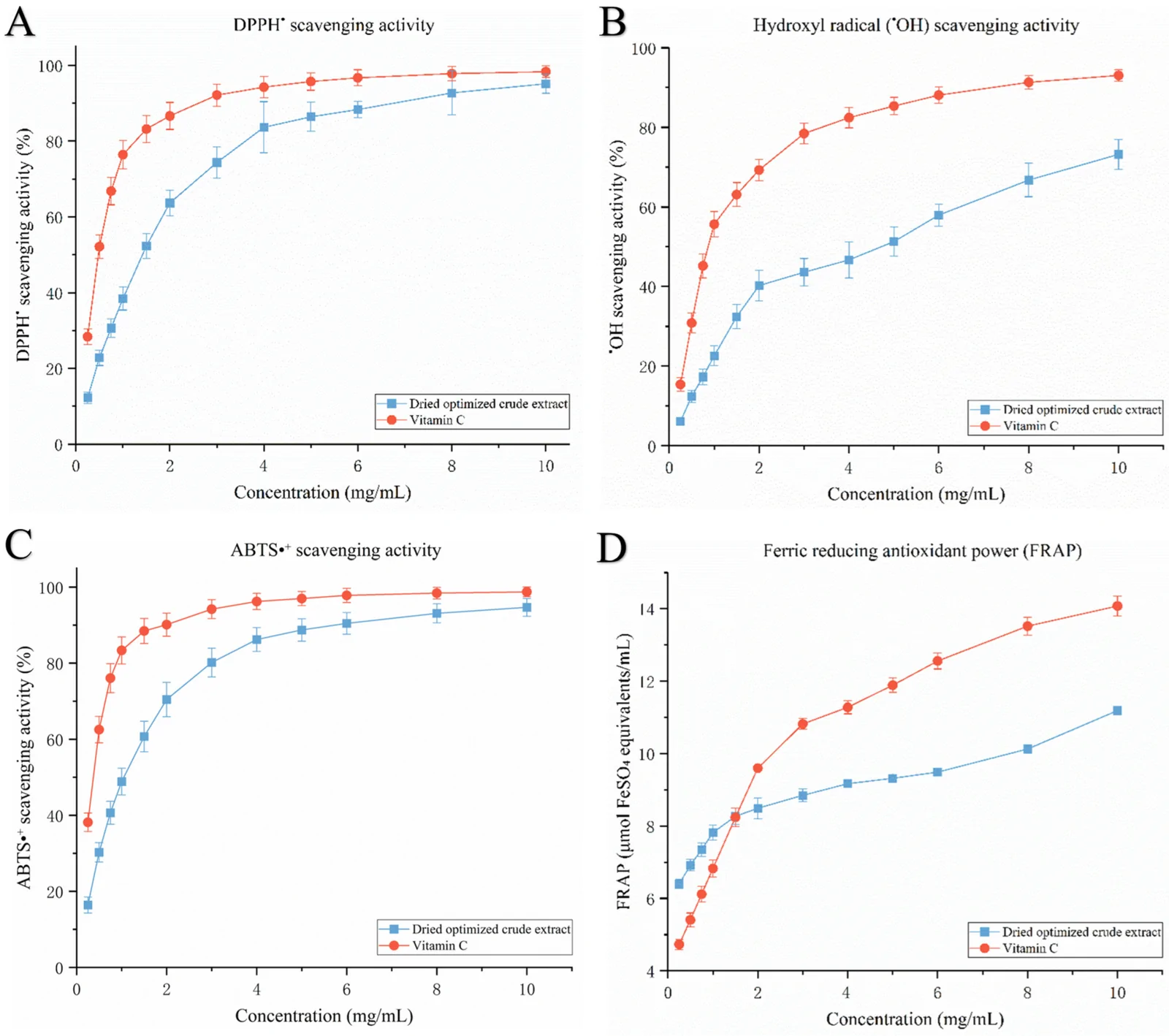
Concentration-dependent antioxidant responses of the dried optimized crude extract and vitamin C. (**A**) DPPH^•^ scavenging activity. (**B**) Hydroxyl radical (^•^OH) scavenging activity. (**C**) ABTS^•+^ scavenging activity. (**D**) Ferric reducing antioxidant power (FRAP), expressed as μmol FeSO_4_ equivalents/mL. Extract concentrations are reported on a dried crude extract mass basis. At each tested concentration, extract values are presented as mean ± SD from three independently prepared dried crude extract batches (*n* = 3), whereas vitamin C values are presented as mean ± SD from the three corresponding analytical runs (*n* = 3).

Antioxidant properties were assessed using assays that capture radical-scavenging and reducing behavior [16,24]. Across the concentration series, the dried optimized crude extract produced progressively stronger DPPH^•^, ^•^OH, and ABTS^•+^ scavenging responses (Figure 3A–C).

Expressed per mass of dried crude extract, the mean IC_50_ values were 1.339 ± 0.061 mg/mL for DPPH^•^, 3.931 ± 0.108 mg/mL for ^•^OH, and 1.004 ± 0.032 mg/mL for ABTS^•+^ (Table 5). Vitamin C reached 50% scavenging at lower concentrations in all three radical-scavenging assays. This comparison should be interpreted cautiously because vitamin C is a purified reference, whereas the extract contains multiple constituents; the reported IC_50_ values describe the whole crude extract rather than purified quercetin.

Quantitative comparison with previous studies provides further context for the antioxidant activity of the present crude extract. Nguyen et al. reported DPPH^•^ and ABTS^•+^ IC_50_ values of 14.91 ± 0.33 and 8.22 ± 0.11 μg/mL, respectively, for a non-roasted methanolic extract of *S. japonica* buds, and 9.31 ± 0.10 and 5.25 ± 0.05 μg/mL, respectively, for a dark-yellow-roasted extract [22]. The corresponding mean IC_50_ values in the present study were 1.339 and 1.004 mg/mL, equivalent to 1339 and 1004 μg/mL, respectively. The lower IC_50_ values reported by Nguyen et al. indicate greater apparent radical-scavenging potency under their specific extraction and assay conditions. Fan et al. reported maximum DPPH^•^ and ABTS^•+^ scavenging activities of 89.29% and 97.86%, respectively, for a hydroalcoholic *S. japonica* flower-bud extract at 1.0 mg/mL [10]. Although these single-concentration scavenging percentages are not equivalent to IC_50_ values, they provide an additional concentration-specific comparison. These cross-study comparisons should be interpreted as contextual rather than as direct rankings of antioxidant potency because extraction solvent and processing, extract composition, concentration basis, sample-to-reagent ratio, reaction conditions, and data-analysis procedures differed among studies. Neither comparator study reported ^•^OH scavenging in a form directly comparable with the present assay, and the FRAP result reported by Fan et al. used a different endpoint; therefore, no numerical comparisons were made for these assays.

FRAP increased across the same concentration range (Figure 3D). At 10 mg/mL, the extract reached 11.19 ± 0.04 μmol FeSO_4_ equivalents/mL, compared with 14.08 ± 0.28 μmol FeSO_4_ equivalents/mL for vitamin C. Quercetin may contribute to this response, but the result more likely reflects the combined redox behavior of phenolic and flavonoid constituents present in the extract [1,2,22].

### 3.4. Antibacterial Activity of the Dried Optimized Crude Extract

**Figure 4:**
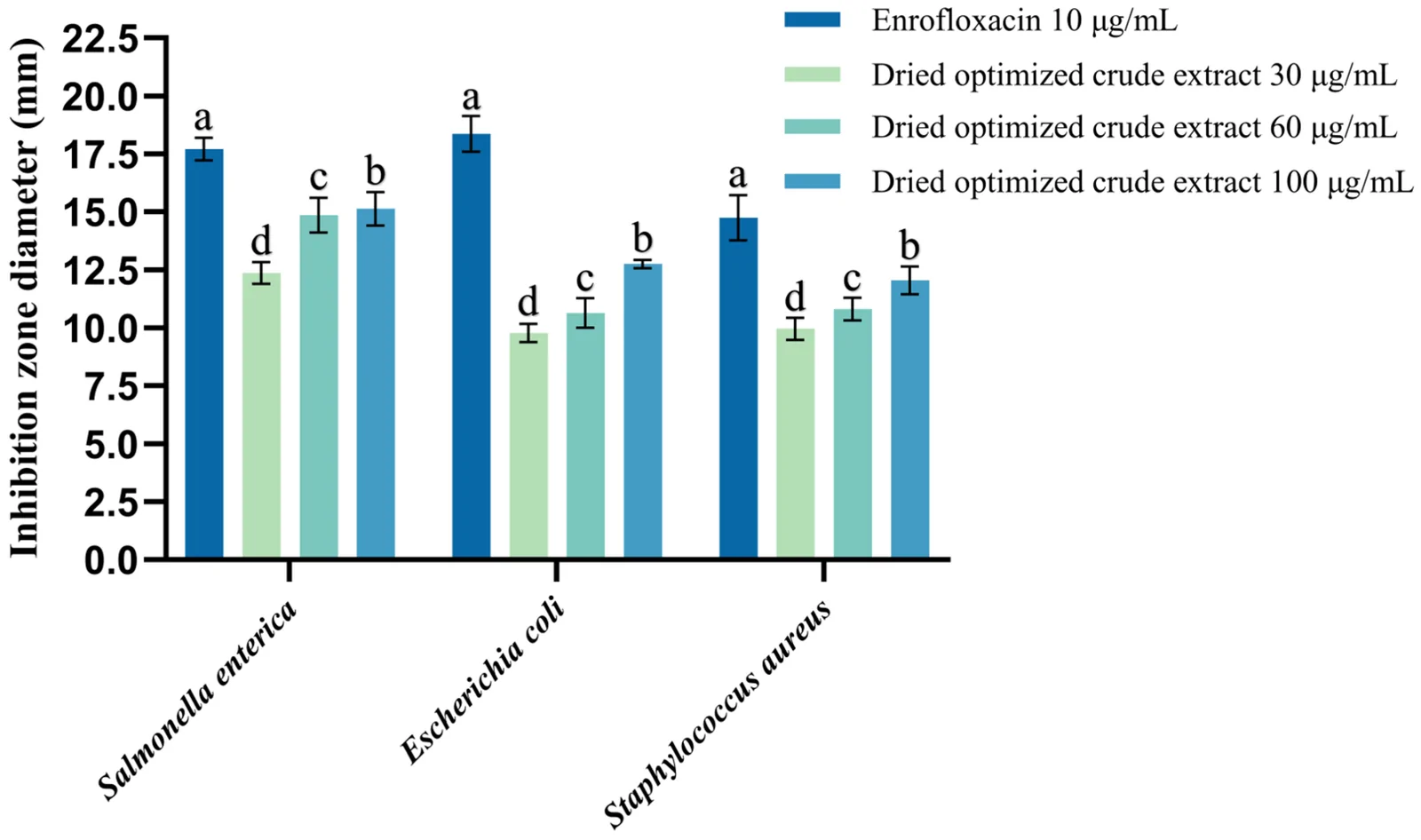
Inhibition zones produced by the dried optimized crude extract against *Salmonella enterica*, *Escherichia coli*, and *Staphylococcus aureus* in the disk-diffusion assay. Enrofloxacin (10 μg/mL; 10 μL/disk, corresponding to 0.1 μg/disk) was used as the positive control. The extract was applied at loading-solution concentrations of 30, 60, and 100 μg/mL using 10 μL/disk, corresponding to 0.3, 0.6, and 1.0 μg dried crude extract per disk, respectively. The absolute-ethanol vehicle control yielded a measured diameter of 6.00 ± 0.00 mm, corresponding to the paper disk alone, and is not displayed. Extract values are presented as mean ± SD from three independently prepared dried crude extract batches (*n* = 3); enrofloxacin was tested in parallel in the corresponding three disk-diffusion runs, and its values are presented as mean ± SD (*n* = 3). Different lowercase letters above the bars within each bacterial strain indicate significant differences among treatments (*p* < 0.05).

All three bacterial strains exhibited measurable inhibition zones in the disk-diffusion assay (Figure 4). The absolute-ethanol vehicle control yielded a measured diameter of 6.00 ± 0.00 mm, corresponding to the paper disk alone, and was therefore omitted from Figure 4. Increasing the loading-solution concentration from 30 to 100 μg/mL, corresponding to 0.3–1.0 μg dried crude extract per disk, increased the inhibition-zone diameter. At the highest loading of 100 μg/mL (1.0 μg/disk), the zones were 15.14 ± 0.94 mm for *S. enterica*, 12.75 ± 0.34 mm for *E. coli*, and 12.04 ± 0.78 mm for *S. aureus*. Enrofloxacin, a purified antibiotic control applied at 10 μg/mL (0.1 μg/disk), produced larger zones. However, zone diameters were not used to infer mass-normalized antibacterial potency relative to enrofloxacin because the crude extract and the purified antibiotic differ substantially in composition and agar diffusivity.

Broth microdilution provided complementary quantitative evidence of antibacterial activity through MIC and MBC determination (Table 6). Disk-diffusion zone diameters and broth-microdilution MIC/MBC values were interpreted as complementary rather than directly numerically comparable endpoints. Agar diffusion is influenced by the mobility and local distribution of extract constituents within the disk–agar system, whereas broth microdilution evaluates bacterial growth inhibition at defined bulk concentrations. *S. aureus* was the most susceptible strain, with MIC and MBC values of 64 and 128 μg/mL, respectively. The corresponding MIC and MBC values were 128 and 256 μg/mL for *S. enterica* and 256 and 512 μg/mL for *E. coli*, respectively. All values are reported on a dried crude extract mass basis. All MBC/MIC ratios were 2, consistent with preliminary bactericidal potential under the present assay conditions. In the 0.512% (*v*/*v*) ethanol vehicle-control wells, visible growth was comparable to that in the corresponding growth controls, and the OD_600_ values did not differ significantly from those of the growth controls (Table S3; *p* > 0.05). Thus, ethanol at the highest final concentration did not account for the observed antibacterial activity.

Variation among the tested organisms may reflect differences in cell-envelope architecture, permeability barriers, efflux systems, and strain-specific susceptibility to phenolic constituents. Quercetin and related flavonoids have been associated with effects on bacterial membrane integrity, enzyme activity, efflux activity, and cellular redox balance [26,27,28]. The lower MIC and MBC values observed for *S. aureus* than for the Gram-negative strains are consistent with the additional permeability barrier imposed by the Gram-negative outer membrane [27,28].

Because the tested material was an unfractionated crude extract, its antibacterial activity should not be attributed to quercetin alone. Quercetin and other co-extracted flavonoid and phenolic constituents may have contributed independently or through combined effects. Such interactions could be additive, synergistic, or antagonistic; however, the present study did not test individual constituents, defined combinations, or chromatographic fractions and therefore provides no direct evidence of synergism. Demonstrating synergistic interactions would require comparison of the crude extract with individual standards and activity-guided fractions, together with combination assays such as checkerboard microdilution and time–kill assays. Accordingly, the current findings should be regarded as preliminary in vitro screening evidence rather than evidence of efficacy in animals or food systems.

### 3.5. Integrated Evaluation of Extraction Efficiency and Bioactivity

Interpretation of the extraction and activity results requires attention to the different analytical bases used in this study. Targeted HPLC estimated the quercetin content of the standardized recovered supernatants, normalized to initial dry plant material, whereas the antioxidant and antibacterial assays used dried crude extracts prepared from the remaining standardized supernatant of each corresponding extraction batch. These different analytical bases preclude direct quantitative comparison between the HPLC-estimated quercetin value and the bioactivity measurements. Accordingly, the observed activities should be attributed to the complete crude extract rather than to quercetin alone.

Quercetin was selected as the chromatographic marker, although the dried product also contained other phenolic and flavonoid constituents. These compounds may contribute individually or collectively to the antioxidant and antibacterial responses. Ultrasound can modify plant tissue structure and facilitate mass transfer, providing a plausible processing context for the extraction pattern observed here [29,30].

Several analytical limitations should also be recognized. The UV response used for process optimization was a quercetin-equivalent signal rather than compound-specific quantification and may have included contributions from other co-extracted UV-absorbing constituents, including flavonoids and other phenolic compounds. Consequently, differences among experimental runs should be interpreted as changes in the overall UV-responsive extractable fraction rather than as changes in quercetin alone. Targeted HPLC estimated quercetin only in recovered supernatants obtained under the optimized extraction condition on the basis of retention-time agreement and external calibration; rutin, intermediate quercetin glycosides, and other co-extracted flavonoids were not quantified, nor were relevant analytes measured separately in the discarded pretreatment liquid and retained solid fraction. Because each independent extraction batch was injected once, the reported SD reflects between-batch variation in the combined extraction-and-HPLC workflow; technical injection repeatability was not assessed separately. TPC and TFC are colorimetric equivalent indices rather than compound-specific measurements. Moreover, the reported μg/mL, mg/mL, mg/g, and percentage values represent different analytical stages or normalization bases, and their conversion depends on the stated dilution factors, calculation volumes, and sample masses. Accordingly, these analytical outputs should be interpreted within their method-specific measurement and normalization frameworks.

Because the dried extract likely contained structurally diverse polyphenols, its antioxidant and antibacterial responses may reflect multiple structural features and biological mechanisms, including hydroxylation, conjugation, metal binding, membrane effects, enzyme inhibition, biofilm modulation, and quorum-sensing interference [31,32,33,34,35,36,37,38,39]. These mechanisms were not directly tested in the present study. Although enzyme-assisted ultrasonication is often discussed within the broader context of green extraction [40], no comparative assessment of solvent consumption, energy use, or environmental impact was performed; therefore, no environmental-performance claim is made. From a process-development perspective, the present laboratory-scale protocol does not yet establish economic feasibility or industrial scalability. At the validated 10.0 g batch scale, the procedure required 150 mL of acetate buffer, 150 mg of enzyme preparations in total, 190 mL of 68% (*v*/*v*) ethanol for ultrasonication, additional 68% (*v*/*v*) ethanol as required to adjust the recovered supernatant to 150 mL, 72 min of enzymatic digestion, two centrifugation steps, solvent evaporation, and drying. These operations identify enzyme input, buffer and water use, ethanol consumption and potential recovery, energy consumption during sonication and temperature control, solid–liquid separation, and drying as potential cost and throughput drivers. Scale-up of ultrasound-assisted extraction is not necessarily linear; a pilot-scale study has shown that maintaining laboratory-scale extraction performance may require adjustment of volumetric ultrasonic power input and the use of appropriately designed continuous-flow reactors [41]. A techno-economic and environmental assessment conducted using another food-derived matrix showed that raw-material and labor costs, electricity consumption, solvent demand, and solvent recovery can materially influence overall process performance and manufacturing costs [42]. Because these variables were not systematically evaluated in the present study, no conclusion can yet be drawn regarding commercial viability. Industrial translation would require pilot-scale validation incorporating mass and energy balances, overall process yield and batch-to-batch consistency, enzyme-use efficiency and ethanol recovery or reuse, continuous or semi-continuous processing, product standardization criteria, and formal techno-economic and life-cycle assessments.

Beyond these analytical and scale-up considerations, the optimized protocol provides a defined route for producing a quercetin-containing crude extract from *S. japonica* flower buds, but its limitations should be acknowledged. The bioactivity tests were confined to in vitro systems, mechanisms were not resolved, and behavior in food matrices, during processing, or during storage was not examined. Future work should prospectively validate dry-extract recovery and batch-to-batch consistency using a predefined mass-recovery protocol and combine untargeted LC–MS/MS profiling with targeted quantification at selected RSM conditions to characterize rutin, quercetin, intermediate quercetin glycosides, and other co-extracted constituents. Such analyses would help distinguish enhanced extraction of pre-existing compounds from enzyme-mediated conversion and identify candidate constituents for subsequent biological validation. Activity-guided fractionation, food-matrix experiments, and stability studies would further strengthen the interpretation and practical relevance of the findings.

## 4. Conclusions

This work developed and validated an enzyme-assisted ultrasonic procedure for *Sophora japonica* flower buds using the UV-estimated quercetin-equivalent yield as the optimization endpoint. At the selected validation conditions—14 U/mL β-glucosidase, 72 min of digestion, and a cellulase-to-pectinase mass ratio of 1.25:1 (*w*/*w*)—the yield reached 7.34%, exceeding the ultrasound-only comparator. Targeted HPLC of the standardized recovered supernatants estimated quercetin at 71.93 ± 1.35 mg/g initial dry plant material. The dried optimized crude extract had total phenolic and flavonoid contents of 82.93 ± 3.75 mg GAE/g and 154.68 ± 5.56 mg QE/g, respectively. It produced concentration-dependent antioxidant responses and showed preliminary in vitro antibacterial activity against *S. enterica*, *E. coli*, and *S. aureus*. The protocol provides a defined route for producing a quercetin-containing crude extract from *S. japonica* flower buds, although the relative contributions of enhanced extraction and enzyme-mediated conversion remain unresolved.

## Figures and Tables

**Table 1:** Box–Behnken design and experimental responses for enzyme-assisted ultrasonic extraction.

Run	A: β-Glucosidase Concentration (U/mL)	B: Digestion Time (min)	C: Cellulase-to-Pectinase Mass Ratio	UV-Estimated Quercetin-Equivalent Yield (%)
1	6	50	1.25	6.58
2	6	90	1.25	6.63
3	14	50	1.25	6.96
4	14	90	1.25	7.00
5	10	50	0.5	6.45
6	10	90	0.5	6.79
7	10	50	2.0	6.57
8	10	90	2.0	6.69
9	6	70	0.5	6.67
10	14	70	0.5	7.04
11	6	70	2.0	6.63
12	14	70	2.0	7.10
13	10	70	1.25	7.31
14	10	70	1.25	7.31
15	10	70	1.25	7.25
16	10	70	1.25	7.33
17	10	70	1.25	7.26

A, B, and C represent the actual factor levels. The response variable was the UV-estimated quercetin-equivalent yield, expressed as % (*w*/*w*) of initial dry plant material. C was defined as the mass of the commercial cellulase preparation divided by that of the commercial pectinase preparation; thus, *C* = 1.25 corresponds to a cellulase-to-pectinase mass ratio of 1.25:1 (*w*/*w*).

**Table 2:** ANOVA and model adequacy of the quadratic model for enzyme-assisted ultrasonic extraction.

Source	Sum of Squares	df	Mean Square	*F*-Value	*p*-Value	Significance
Model	1.49	9	0.1653	50.3	<0.0001	**
A: β-Glucosidase concentration	0.316	1	0.316	96.16	<0.0001	**
B: Digestion time	0.0378	1	0.0378	11.51	0.0116	*
C: Cellulase-to-pectinase mass ratio	0.0002	1	0.0002	0.0609	0.8122	NS
AB	0.0	1	0.0	0.0076	0.9329	NS
AC	0.0025	1	0.0025	0.7607	0.4120	NS
BC	0.0121	1	0.0121	3.68	0.0965	NS
A^2^	0.0736	1	0.0736	22.41	0.0021	**
B^2^	0.5679	1	0.5679	172.8	<0.0001	**
C^2^	0.3783	1	0.3783	115.11	<0.0001	**
Residual	0.023	7	0.0033			
Lack of fit	0.0181	3	0.006	4.95	0.0781	NS
Pure error	0.0049	4	0.0012			
Cor total	1.51	16				

*R*^2^ = 0.9848; adjusted *R*^2^ = 0.9652; predicted *R*^2^ = 0.8030; C.V. = 0.83%. ** *p* < 0.01; * *p* < 0.05; NS, not significant.

**Table 3:** Validation of optimized extraction conditions and comparison of extraction methods.

Section	Method	Optimized/Validated Condition	Predicted Yield (%)	Experimental Yield (%)	Relative Error (%)	*p*-Value
Validation	Ultrasound-only extraction	68% ethanol; 14 min; 19 mL/g liquid-to-material ratio	4.19	4.17	0.48	
Validation	Enzyme-assisted ultrasonic extraction	14 U/mL β-glucosidase; 72 min digestion; cellulase-to-pectinase mass ratio of 1.25:1 (*w*/*w*)	7.36	7.34	0.30	
Comparison	Ultrasound-only extraction	Validated optimized condition		4.17 ± 0.22 ^b^		<0.001
Comparison	Enzyme-assisted ultrasonic extraction	Validated optimized condition		7.34 ± 0.45 ^a^		<0.001

Predicted and experimental values in this table are UV-estimated quercetin-equivalent yields expressed as % (*w*/*w*) of initial dry plant material. For method comparison, values are expressed as mean ± SD of three independent extraction experiments (*n* = 3). Values with different superscript letters indicate significant differences (*p* < 0.05). Relative errors were calculated using unrounded model-predicted values.

**Table 4:** HPLC quantification of recovered supernatants and phytochemical characterization of the dried optimized crude extract.

Parameter	Standard/Method	Linear Range	Regression Equation	*R* ^2^	Result
HPLC calibration curve	Quercetin	10–100 μg/mL	*y* = 42,495.1*x* + 9012.9	0.9999997	—
Retention time of quercetin standard	HPLC	—	—	—	8.22 min
Retention-time range of assigned quercetin peaks in recovered supernatants	HPLC	—	—	—	8.23–8.24 min
Mean peak area of assigned quercetin peaks in injected solutions	HPLC	—	—	—	3,065,654 ± 57,187
Quercetin concentration in injected solutions	HPLC	—	—	—	71.93 ± 1.35 μg/mL
Quercetin concentration in recovered supernatants	HPLC	—	—	—	4.795 ± 0.090 mg/mL
Quercetin content relative to initial dry plant material	HPLC	—	—	—	71.93 ± 1.35 mg/g initial dry plant material
HPLC-estimated quercetin yield relative to initial dry plant material	HPLC	—	—	—	7.19 ± 0.13%
Total phenolic content (TPC)	Gallic acid	0–18 μg/mL	*A* = 0.032262*C*_GA_ − 0.0012	0.9999	82.93 ± 3.75 mg GAE/g dried crude extract
Total flavonoid content (TFC)	Quercetin	0–7 μg/mL	*A* = 0.0699*C*_QE_ − 0.0243	0.9981	154.68 ± 5.56 mg QE/g dried crude extract

For HPLC, *y* represents peak area and *x* represents the quercetin concentration in the injected solution (μg/mL). The concentration in the recovered supernatant was obtained by multiplying the injected-solution concentration by the 66.67-fold dilution factor and converting μg/mL to mg/mL. Quercetin content and yield were calculated relative to the initial dry plant material. For TPC and TFC, *A* represents absorbance, whereas *C*_GA_ and *C*_QE_ represent the calibration-derived gallic acid-equivalent and quercetin-equivalent concentrations, respectively, of the diluted sample solutions (μg/mL). TPC and TFC were calculated using Equations (4) and (5), respectively, with correction for the initial sample solution volume, total pre-assay dilution factor, and dried crude extract mass. GAE, gallic acid equivalents; QE, quercetin equivalents. Quantitative HPLC results are expressed as mean ± SD of three independently prepared extraction batches (*n* = 3), with each batch treated as the experimental unit and injected once. Batch-specific peak areas, concentrations, and mass-normalized contents were calculated separately before being summarized as mean ± SD. Retention times for the recovered supernatants from the optimized extraction batches are reported as the observed range across the three batches.

**Table 5:** Antioxidant capacity of the dried optimized crude extract and vitamin C.

Assay	Parameter	Dried Crude Extract	Vitamin C
DPPH^•^	IC_50_ (mg/mL)	1.339 ± 0.061	0.468 ± 0.031
^•^OH	IC_50_ (mg/mL)	3.931 ± 0.108	0.942 ± 0.018
ABTS^•+^	IC_50_ (mg/mL)	1.004 ± 0.032	0.344 ± 0.013
FRAP	Maximum value in tested range	11.19 ± 0.04 μmol FeSO_4_ equivalents/mL at 10.0 mg/mL	14.08 ± 0.28 μmol FeSO_4_ equivalents/mL at 10.0 mg/mL

For each tested concentration, extract scavenging activities are presented as mean ± SD from three independently prepared dried crude extract batches (*n* = 3), whereas vitamin C values are presented as mean ± SD from the three corresponding analytical runs (*n* = 3). For each of the DPPH^•^, ^•^OH, and ABTS^•+^ assays, the concentration-response dataset from each independent dried crude extract batch was fitted separately using a two-parameter Hill model, with the lower and upper asymptotes constrained to 0% and 100%, respectively. The three corresponding vitamin C datasets were fitted separately using the same model. IC_50_ values are presented as mean ± SD (*n* = 3 independent extraction batches for the dried crude extract and *n* = 3 corresponding analytical runs for vitamin C). IC_50_, half-maximal inhibitory concentration. Concentrations of the dried optimized crude extract are expressed on a dried crude extract mass basis. FRAP was expressed as μmol FeSO_4_ equivalents/mL.

**Table 6:** MIC and MBC of the dried optimized crude extract against tested bacterial strains.

Bacterial Strain	MIC (μg/mL Dried Crude Extract)	MBC (μg/mL Dried Crude Extract)	MBC/MIC Ratio	Interpretation
*Staphylococcus aureus*	64	128	2	Preliminary bactericidal potential
*Salmonella enterica*	128	256	2	Preliminary bactericidal potential
*Escherichia coli*	256	512	2	Preliminary bactericidal potential

An MBC/MIC ratio of 4 or lower was treated as a preliminary indicator of bactericidal potential under these assay conditions [25]. MIC and MBC are reported on a dried crude extract basis. The 0.512% (*v*/*v*) ethanol vehicle control did not differ from the matched growth control for any strain (Table S3; *p* > 0.05). Broth-microdilution assays were independently performed using three dried crude extract batches. Identical MIC and MBC endpoints were obtained across the three independent batches.

## Data Availability

The original contributions presented in the study are included in the article/Supplementary Material, further inquiries can be directed to the corresponding authors.

## Supplementary Materials

The following supporting information can be downloaded at: https://www.mdpi.com/article/10.3390/foods15162781/s1, Figure S1: Model-based response surfaces and contour plots for ultrasound-only extraction of Sophora japonica flower buds. (A,B) Effects of ethanol concentration and ultrasonication time at a fixed liquid-to-material ratio of 20 mL/g. (C,D) Effects of ethanol concentration and liquid-to-material ratio at a fixed ultrasonication time of 15 min. (E,F) Effects of ultrasonication time and liquid-to-material ratio at a fixed ethanol concentration of 70% (*v*/*v*). The response variable was the UV-estimated quercetin-equivalent yield (%).; Figure S2: HPLC chromatograms of recovered supernatants from three independent extraction batches prepared under the validated enzyme-assisted ultrasonic extraction condition. (A–C) Full chromatograms of extraction batches 1–3, respectively. The assigned quercetin peaks eluted at 8.23, 8.24, and 8.24 min, respectively. The vertical dashed lines in panels A–C indicate the retention times of the assigned quercetin peaks in extraction batches 1–3 (8.23, 8.24, and 8.24 min, respectively). (D) Overlay of the assigned quercetin peak region (7.80–8.60 min) for the three batches. Peak assignment was based on retention-time agreement with the quercetin reference standard at 8.22 min, and quantification was performed using the external calibration curve shown in Figure 2C. Table S1: Box–Behnken design and experimental UV-estimated quercetin-equivalent yields for ultrasound-only extraction. Table S2: Analysis of variance and model adequacy of the quadratic model for ultrasound-only extraction; Table S3: Optical density at 600 nm (OD_600_) of the 0.512% (*v*/*v*) ethanol vehicle control in the broth microdilution assay.
