# Supplementary material for: Optimization of Enzyme-Assisted Ultrasonic Extraction of a Quercetin-Containing Crude Extract from Sophora japonica Flower Buds and Evaluation of Its In Vitro Antioxidant and Antibacterial Activities
Source: Foods. 2026 Aug 7;15(16):2781. doi: 10.3390/foods15162781 (PMC13512480; doi:10.3390/foods15162781)
Supplement: Supplementary file 1 [file foods-15-02781-s001.zip › foods-4420778-supplementary.pdf]

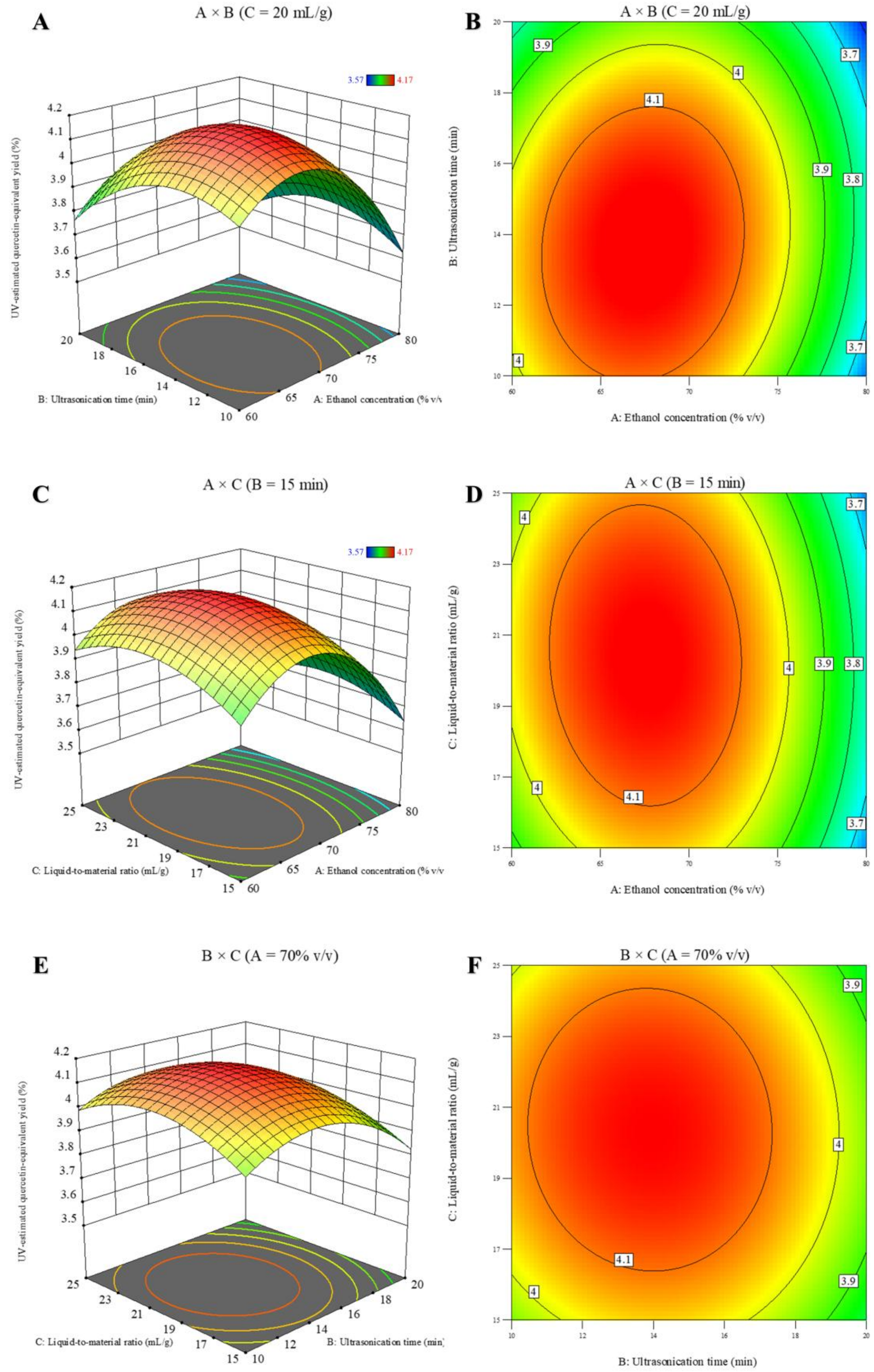

Figure S1. Model-based response surfaces and contour plots for ultrasound-only extraction of *Sophora japonica* flower buds. (A,B) Effects of ethanol concentration and ultrasonication time at a fixed liquid-to-material ratio of 20 mL/g. (C,D) Effects of ethanol concentration and liquid-to-material ratio at a fixed ultrasonication time of 15 min. (E,F) Effects of ultrasonication time and liquid-to-material ratio at a fixed ethanol concentration of 70% (v/v). The response variable was the UV-estimated quercetin-equivalent yield (%).

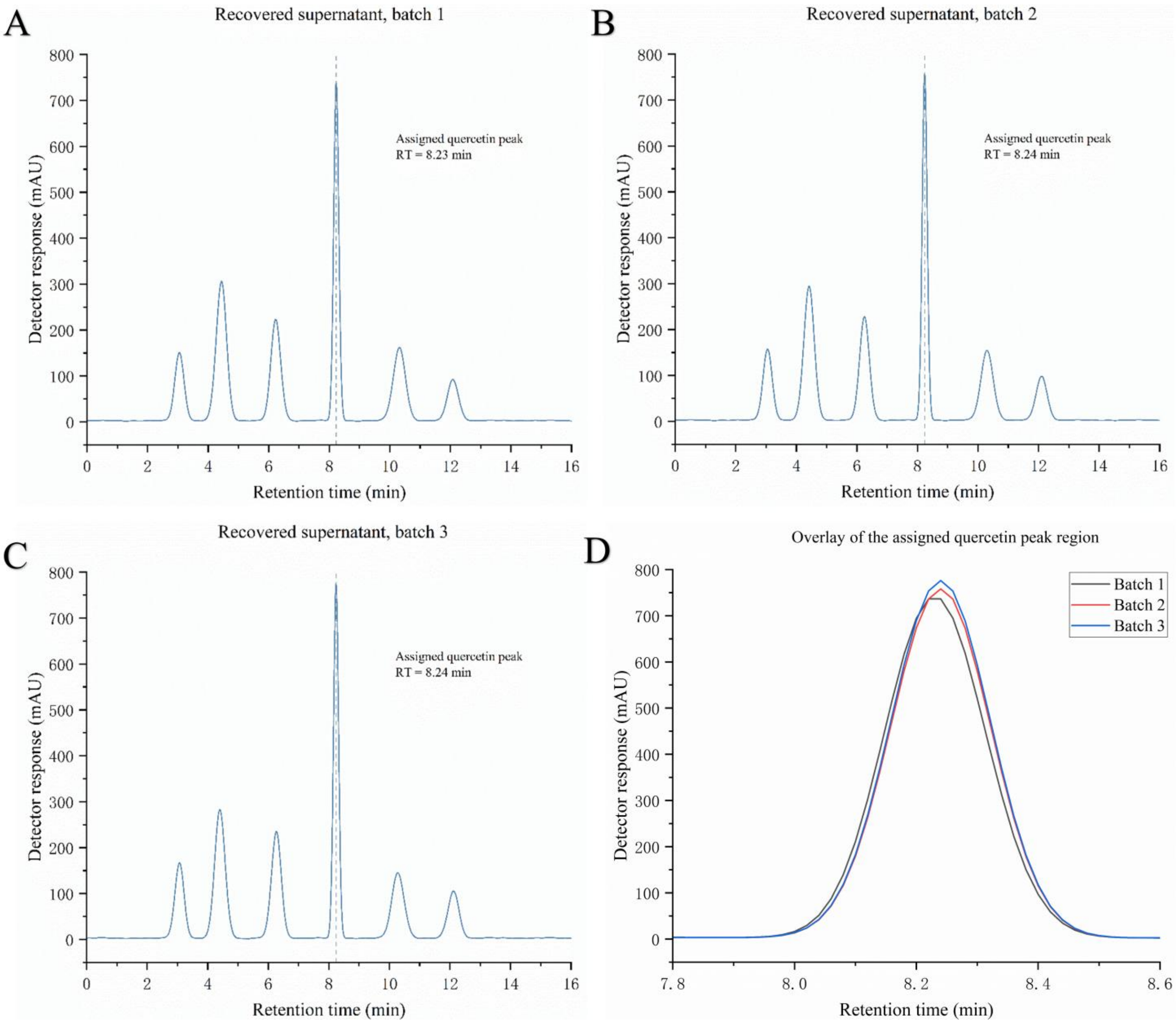

Figure S2. HPLC chromatograms of recovered supernatants from three independent extraction batches prepared under the validated enzyme-assisted ultrasonic extraction condition. (A–C) Full chromatograms of extraction batches 1–3, respectively. The assigned quercetin peaks eluted at 8.23, 8.24, and 8.24 min, respectively. The vertical dashed lines in panels A–C indicate the retention times of the assigned quercetin peaks in extraction batches 1–3 (8.23, 8.24, and 8.24 min, respectively). (D) Overlay of the assigned quercetin peak region (7.80–8.60 min) for the three batches. Peak assignment was based on retention-time agreement with the quercetin reference standard at 8.22 min, and quantification was performed using the external calibration curve shown in Figure 2C.

Table S1. Box–Behnken design and experimental UV-estimated quercetin-equivalent yields for ultrasound-only extraction.

Run	*A*: Ethanol concentration (%)	*B*: Ultrasonication time (min)	*C*: Liquid-to-material ratio (mL/g)	UV-estimated quercetin-equivalent yield (%)
1	70	20	25	3.84
2	80	15	20	3.77
3	60	20	20	3.76
4	70	15	20	4.17
5	70	10	15	3.95
6	70	15	20	4.15
7	60	15	25	3.95
8	70	15	20	4.17
9	80	15	15	3.63
10	80	20	20	3.57
11	60	10	20	3.98
12	70	15	20	4.16
13	70	10	25	3.98
14	80	15	25	3.64
15	70	20	15	3.84
16	70	15	20	4.16
17	60	15	15	3.87

The response variable was the UV-estimated quercetin-equivalent yield (%). The ultrasound-only procedure served as the nonenzymatic comparative extraction method.

Table S2. Analysis of variance and model adequacy of the quadratic model for ultrasound-only extraction.

Source	Sum of squares	df	Mean square	*F*-value	*p*-value	Significance
Model	0.6491	9	0.0721	409.61	<0.001	**
A: Ethanol concentration	0.1215	1	0.1215	690.19	<0.001	**
B: Ultrasonication time	0.0244	1	0.0244	138.71	<0.001	**
C: Liquid-to-material ratio	0.0018	1	0.0018	10.22	0.0151	*
AB	0.0036	1	0.0036	20.39	0.0027	**
AC	0.0012	1	0.0012	6.96	0.0335	*
BC	0.0002	1	0.0002	1.28	0.2955	NS
A^2	0.2971	1	0.2971	1687.49	<0.001	**
B^2	0.0758	1	0.0758	430.55	<0.001	**
C^2	0.0478	1	0.0478	271.64	<0.001	**
Residual	0.0012	7	0.0002			
Lack of fit	0.0010	3	0.0003	4.54	0.0891	NS
Pure error	0.0003	4	0.0001			
Cor total	0.6504	16				

R^2 = 0.9981; adjusted R^2 = 0.9957; predicted R^2 = 0.9600; C.V. = 0.34%. ** $p < 0.01$; * $p < 0.05$; NS, not significant. The lack of fit was not significant (p = 0.0891).

Table S3. Optical density at 600 nm (OD_{600}) of the 0.512% (v/v) ethanol vehicle control in the broth microdilution assay.

Bacterial strain	Sterility control (OD_{600})	Growth control (OD_{600})	0.512% ethanol vehicle control (OD_{600})	*p*-value vs. growth control
Salmonella enterica	0.04 ± 0.01	0.81 ± 0.02	0.78 ± 0.03	> 0.05
Escherichia coli	0.05 ± 0.01	0.83 ± 0.02	0.80 ± 0.02	> 0.05
Staphylococcus aureus	0.05 ± 0.01	0.86 ± 0.02	0.82 ± 0.02	> 0.05

OD_{600}, optical density measured at 600 nm. Values are presented as mean ± SD ($n = 3$). The 0.512% ethanol vehicle control did not differ significantly from the corresponding growth control for any tested strain ($p > 0.05$).
